# Effect of Pilates on Glucose and Lipids: A Systematic Review and Meta-Analysis of Randomized Controlled Trials

**DOI:** 10.3389/fphys.2021.641968

**Published:** 2021-05-28

**Authors:** Zehua Chen, Xiangling Ye, Yubo Xia, Huiting Song, Yi Wang, Yingxin Guan, Zhen Shen, Weijian Chen, Tao Jiang, Huai Wu, Xuemeng Xu

**Affiliations:** ^1^The Fifth Clinical Medical School, Guangzhou University of Chinese Medicine, Guangzhou, China; ^2^Kunming Municipal Hospital of Traditional Chinese Medicine, The Third Affiliated Hospital of Yunnan University of Chinese Medicine, Kunming, China; ^3^Jiangxi Academy of Traditional Chinese Medicine, Nanchang, China; ^4^Guangdong Second Traditional Chinese Medicine Hospital, Guangzhou, China

**Keywords:** Pilates, exercise, glucose, lipids, meta-analysis, review

## Abstract

**Objective:** The benefits of Pilates for blood glucose and lipids remain unclear. The purpose of this study was to examine the effect of Pilates on their levels.

**Methods:** Searches were conducted in five databases to identify relevant articles published until October 29, 2020. Paired reviewers independently screened the articles and extracted data from each included study. Meta-analysis was performed to assess the effects of Pilates on blood glucose and lipids. Subgroup analyses and sensitivity analyses were conducted to explore heterogeneity.

**Results:** According to the inclusion and exclusion criteria, 15 randomized controlled trials (RCTs) comprising 587 participants were included in the study. Overall, the Pilates group (PG) had a significantly greater reduction in post-prandial blood glucose than the control group (CG) (MD = −22.25 mg/dL, 95% CI: [−28.34, 16.17] mg/dL, *P* < 0.00001, I^2^ = 0%); glycated hemoglobin (HbA1c) (MD = −0.78%, 95% CI: [−1.13, −0.42]%, *P* < 0.0001, I^2^ = 88%); total cholesterol (TC) (MD = −20.90 mg/dL, 95% CI: [−37.21, −4.60] mg/dL, *P* = 0.01, I^2^ = 84%); triglycerides (TG) (MD = −12.59 mg/dL, 95% CI: [−19.88, −5.29] mg/dL, *P* = 0.0007, I^2^ = 86%); and low density lipoprotein cholesterol (LDL-C) (MD = −12.39 mg/dL, 95% CI: [−16.82, −7.95] mg/dL, *P* < 0.00001, I^2^ = 45%) compared to CG, whereas no significant difference was detected between the two groups in fasting blood glucose (MD = −7.04 mg/dL, 95% CI: [−17.26, 3.17] mg/dL, *P* = 0.18, I^2^ = 93%), insulin (MD = −1.44 μU/mL, 95% CI: [−4.30, 1.41] μU/mL, *P* = 0.32, I^2^ = 0%); and high density lipoprotein cholesterol (HDL-C) (MD = −2.68 mg/dL, 95% CI: [−9.03, 3.67] mg/dL, *P* = 0.41, I^2^ = 89%). However, by subgroup analysis, we found that compared to the CG, PG showed no significant improvement in blood glucose and lipids levels for non-diabetics, while it presented a significantly greater decrease in post-prandial blood glucose, TC, TG, and LDL-C for diabetic patients. Notably, for diabetic patients, Pilates and medication treatments showed no significant reduction in fasting blood glucose (MD = −7.00 mg/dL, 95% CI: [−26.06, 12.06] mg/dL, *P* = 0.40) and HbA1c (MD = −0.23%, 95% CI: [−0.58, 0.13]%, *P* = 0.21, I^2^ = 0%) than medications treatment used alone, and Pilates combined with medications and dietary treatments presented no significant improvement in fasting blood glucose than a combination of medications and dietary treatments (MD = −10.90 mg/dL, 95% CI: [−32.35, 10.54] mg/dL, *P* = 0.32, I^2^ = 94%).

**Conclusions:** Overall, Pilates could improve post-prandial blood glucose, fasting blood glucose, HbA1c, TG, TC, and LDL-C for diabetic patients, which could be influenced by its duration and intensity. Moreover, it had no significant effect on blood glucose and lipids for non-diabetic individuals. However, Pilates, as an adjunctive treatment to medications was not superior to medications used alone in lowering fasting blood glucose and HbA1c. Furthermore, Pilates combined with medications and dietary treatments showed no significant improvement in fasting blood glucose, whereas it had a greater reduction in post-prandial blood glucose and HbA1c for diabetic patients.

**Systematic Review Registration:** https://osf.io/xgv6w.

## Introduction

Lipids and glucose, the main energy sources, are viewed as the pivotal components of organic metabolism in mammals (Guo et al., 2017). Metabolism can be affected by many factors including age, weight, diet, and exercise. Unfavorable alterations in metabolism may result in clinical disorders, such as diabetes and cardiovascular diseases. With the growth of the aging and obese population, more and more people will suffer from hyperglycemia or hyperlipidemia caused by the abnormal metabolism of lipids and glucose. Obviously, hyperglycemia and hyperlipidemia are global health concerns (Aune et al., 2015). Therefore, it is critical and urgent to explore effective and feasible methods to solve this serious problem. Despite the fact that many pharmacological approaches have been developed, it seems to be more acceptable to find a non-pharmacological method to improve glucose and lipids metabolism because of fewer side effects.

As was previously reported, exercise improved insulin resistance (Sampath Kumar et al., 2019), and caused a statistically significant reduction in blood glucose and lipid levels (Nidhi et al., 2012). Hence exercise has attracted a lot of attention to lower the concentration of lipids and glucose in the blood. In recent years, Pilates has been increasingly applied to blood lipids and glucose control. Pilates exercise originated in 1880 (Shand, 2004), aiming to strengthen the body's core muscles (Panhan et al., 2020). According to a previously published meta-analysis, it was proved to be beneficial in many aspects, for instance, improving cardiorespiratory fitness (Fernández-Rodríguez et al., 2019), reducing pain and disability in subjects affected by chronic lower back pain (Miyamoto et al., 2013), and increasing balance in older adults (Casonatto and Yamacita, 2020). Moreover, the benefits of Pilates were detected not only in the individuals with specific disorders but also in the healthy population (Campos et al., 2016). Interestingly, some studies (Zandi et al., 2016; Suna and Kenan, 2020) highlighted that Pilates exerted positive impacts on blood glucose and lipid levels. On the contrary, some other studies (Hagner-Derengowska et al., 2015; Akbas, 2017) suggested that there was insufficient evidence to support the idea that Pilates exercise was a useful strategy for improving glucose and lipids metabolism. Thus, until now, the clinical data estimating the effects of Pilates on glucose and lipid are inconsistent, and there is no conclusive evidence as to whether Pilates is worth promoting for decreasing blood glucose and lipids levels. Consequently, in this study, we aimed to conduct a systematic review and meta-analysis of randomized controlled trials (RCTs) to evaluate the efficacy of Pilates on changes in blood glucose and lipids, which would offer a new insight and further provide reference for the treatment and prevention of diseases resulted in the pathobolism of lipids and glucose in clinical practice.

## Methods

This meta-analysis was conducted in accordance with the guidelines of the Preferred Reporting Items for Systematic Review and Meta-Analyses (PRISMA) (Moher et al., 2009). The protocol of this review is registered at the Open Science Framework (OSF, https://osf.io/xgv6w), and the registration DOI of this study is 10.17605/OSF.IO/XGV6W.

### Search Strategy

The electronic databases of PubMed, EMBASE, Web of science, **CINAHL**, and China National Knowledge Infrastructure (CNKI) were searched until October 29, 2020. MeSH terms and keywords, such as “Exercise Movement Techniques,” “Pilates,” “Pilates-based exercise,” “Training, Pilates,” and “randomized controlled trial,” etc. were used to search without restrictions with respect to language and publication date. The detailed search strategy is documented in Supplementary Figure 1. Additionally, we also used Google Scholar to identify relevant full-text articles.

### Selection Criteria

The selection of studies was performed independently by two researchers. Based on the PICOS approach, we included all the studies which were eligible using the following criteria: ① Patients: without restriction; ② intervention: Pilates; ③ comparators: Pilates vs. other treatments, Pilates + other treatments vs. other treatments, Pilates vs. non-intervention; ④ outcomes: more than one explicitly reported outcome data regarding glucose and lipids levels; ⑤ study design: clinical randomized controlled study; ⑥ languages: published in Chinese or English in a peer-reviewed journal. Studies would be excluded if they met any of the following criteria: ① Conference abstracts, full-text unavailable articles, or unpublished literatures; ② duplicate reports, animal experimental studies, protocol, comments, meta-analysis, or reviews.

### Data Extraction

Two independent investigators (ZC and ZS) screened all the literature in this study. Firstly, we removed all duplicates and then preliminarily selected the articles by reading the titles and abstracts. Secondly, after reading the full text of the remaining studies, we screened them strictly according to the inclusion and exclusion criteria. Finally, we extracted the data in the included literatures without controversy. Main information comprising authors' names, publication year, country, age, weight, body mass index, population group, sample, intervention type, study design, intervention dose, and main outcomes in the included articles were collected carefully. During the period of screening and data extraction, a discrepancy would be resolved through discussion, or consultation with the primary reviewer.

### Risk of Bias and Publication Bias Assessment

Two investigators (ZC and ZS) assessed risk of bias according to the Cochrane Collaboration risk of bias table (Higgins et al., 2011). The risk of bias is assessed from seven aspects: sequence generation, allocation concealment, blind of participants and personnel, blind of outcome, incomplete outcome data, selective reporting, and other biases. The risk of each item is categorized into three levels: high, unclear, and low. We also determined the publication bias by using Begg's test and Egger's test (Shen et al., 2019).

### Statistical Analysis

We performed data analysis by using review manager 5.3 software provided by the Cochrane library for the included studies, and the results were depicted by the forest map intuitively. Begg's test and Egger's test were calculated using Stata 14 (USA, Stata Corp LP, 2015) to evaluate the publication bias. In this study, all parameters were continuous variables. They were pooled by mean differences (MDs) with 95% confidence intervals (95% CI). Cochran Q-test and I^2^ index were employed to analyze the heterogeneity among the studies (Huedo-Medina et al., 2006). An I^2^ statistic > 50% was considered to be substantially heterogeneous. Based on the Cochrane Handbook for Systematic Reviews of Interventions (Higgins and Green, 2011), if heterogeneity was not significantly (I^2^ <50%) observed, fixed effects models were selected; otherwise a random-effects model, sensitivity analyses, or subgroup analyses should be utilized (I^2^>50%). Begg's and Egger's tests were employed to assess publication bias (Moher et al., 2009). The difference was considered statistically significant when *P* < 0.05.

## Results

### Study Selection

**Figure 1:**
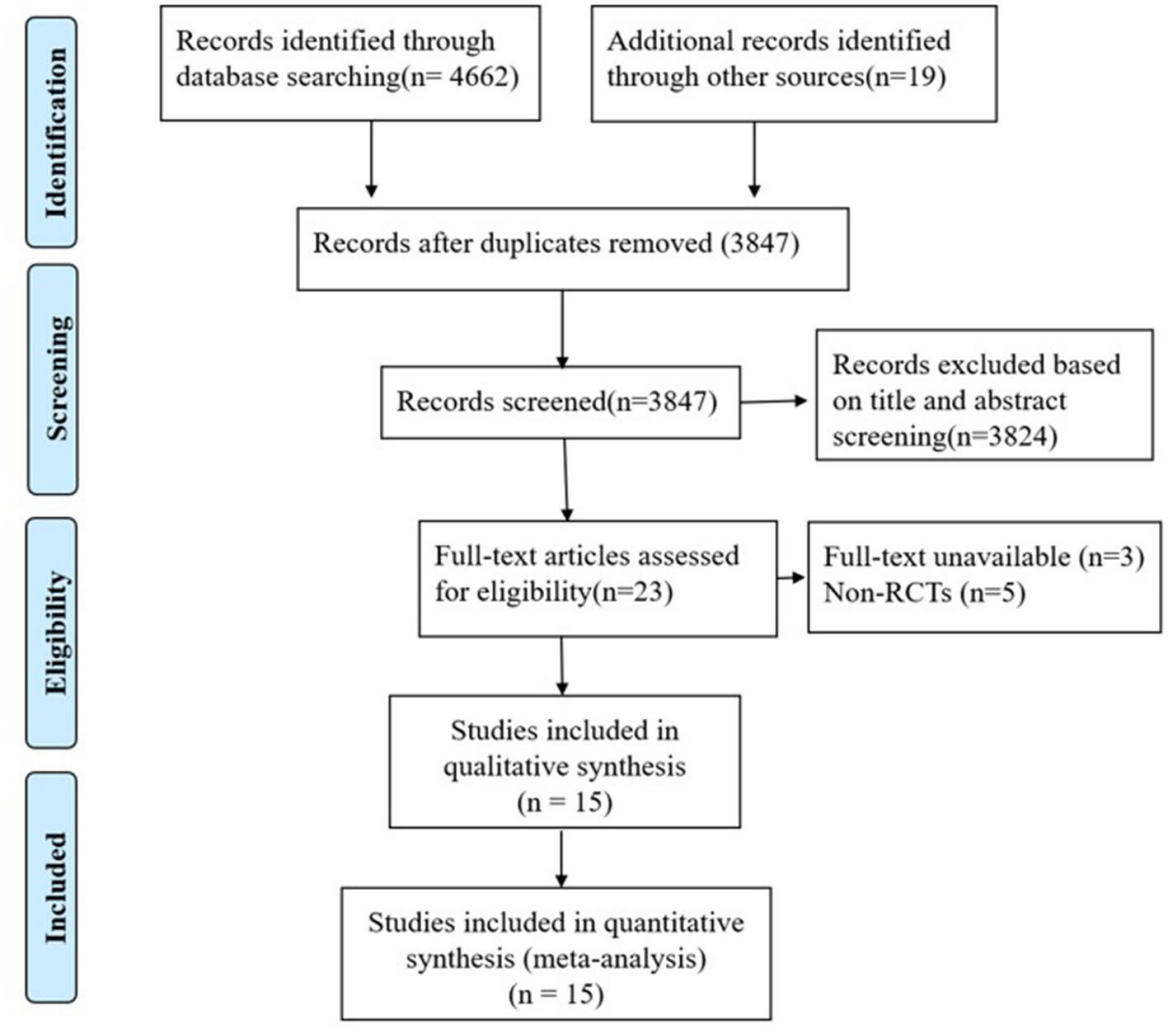
Flowchart of study selection.

In total 4,681 records were identified as potentially relevant by database searching. All the articles were imported into EndNote X8 (Bld, 10063) to remove duplicates. After removing 834 duplicates and eliminating 3,819 articles through screening their titles and abstracts, 15 RCTs (Ramezankhany et al., 2011; Tunar et al., 2012; Marinda et al., 2013; Zolfaghari et al., 2015; Gokgul and Hazar, 2017; Khormizi and Azarniveh, 2017; Yucel and Uysal, 2018; Abd EL-Monim et al., 2019; Aslan et al., 2019; Kumar, 2019; Zhang and Chen, 2019; Batar et al., 2020; Cui et al., 2020; Jung et al., 2020; Melo et al., 2020) comprising 587 participants were reviewed (Figure 1). Thirteen studies were excluded: three without full-text and five non-RCTs (as listed in Supplementary Figure 2). Eight studies (Ramezankhany et al., 2011; Tunar et al., 2012; Marinda et al., 2013; Gokgul and Hazar, 2017; Yucel and Uysal, 2018; Abd EL-Monim et al., 2019; Zhang and Chen, 2019; Batar et al., 2020; Jung et al., 2020; Melo et al., 2020) reported the effects of Pilates on blood lipids, while thirteen studies (Tunar et al., 2012; Marinda et al., 2013; Zolfaghari et al., 2015; Khormizi and Azarniveh, 2017; Yucel and Uysal, 2018; Abd EL-Monim et al., 2019; Aslan et al., 2019; Kumar, 2019; Zhang and Chen, 2019; Batar et al., 2020; Cui et al., 2020; Jung et al., 2020; Melo et al., 2020) recorded its effects on glucose metabolism. Among the included studies, thirteen (Tunar et al., 2012; Zolfaghari et al., 2015; Khormizi and Azarniveh, 2017; Yucel and Uysal, 2018; Abd EL-Monim et al., 2019; Zhang and Chen, 2019; Batar et al., 2020; Cui et al., 2020; Melo et al., 2020) of them assessed the effects of Pilates for patients with diabetes, and four (Khormizi and Azarniveh, 2017; Abd EL-Monim et al., 2019; Aslan et al., 2019; Jung et al., 2020) of them estimated the effects for obese subjects. The characteristics of each included study are summarized in Table 1.

Table 1Characteristics of the included studies.

**Table:** 

**References**	**Country**	**Age (y)**		**Weight (kg)**		**BMI (kg.m**^******−**2****^**)**		**Sample size**	**Drop out**
		**EG**	**CG**	**EG**	**CG**	**EG**	**CG**	**(PG/CG)**	**(PG/CG)**
Marinda et al. (2013)	South Africa	66.12 ± 4.77	65.32 ± 5.01	71.71 ± 14.92	75.19 ± 14.78	28.32 ± 6.77	29.32 ± 5.44	25/25	0/0
Aslan et al. (2019)	Turkey	35.27 ± 9.32	34 ± 9.09	81.58 ± 15.89	77.30 ± 13.24	30.33 ± 6.15	28.76 ± 4.37	29/32	0/0
Ramezankhany et al. (2011)	*Iran*	37.16 ± 2.88	36.5 ± 2.99	73.08 ± 9.16	76.38 ± 8.92	29.11 ± 3.014	31.61 ± 2.90	12/10	0/0
Melo et al. (2020)	Brazil	65.5 ± 5.5	67.5 ± 6.3	66.2 ± 5.4	73.5 ± 6.1	27.6 ± 2.6	30.8 ± 6.0	11/11	0/0
Yucel and Uysal (2018)	Turkey	58.50 ± 7.00	53.50 ± 9.00	NA	NA	32.20 ± 6.93	30.84 ± 8.09	24/21	4/7
Jung et al. (2020)	Korea	43.8 ± 8.6	51.6 ± 6.5	65.8 ± 11.0	60.5 ± 6.8	25.1 ± 3.3	25.2 ± 2.0	10/10	2/2
Tunar et al. (2012)	Turkey	14.2 ± 2.2	14.3 ± 1.8	NA	NA	NA	NA	17/14	0/0
Abd EL-Monim et al. (2019)	Egypt	46 ± 5.15	44.65 ± 6.31	85.35 ± 3.4	86.02 ± 3.52	33.32 ± 1.29	33.49 ± 1.53	20/20	0/0
Gokgul and Hazar (2017)	*Turkey*	22–55	60.66 ± 6.49	68.99 ± 13.63	22.86 ± 3.70	25.74 ± 4.57	11/11	1/1	
Zolfaghari et al. (2015)	Iran	47.92 ± 0.76	48.00 ± 0.63	75.03 ± 3.57	77.75 ± 1.09	30.28 ± 1.37	31.47 ± 0.41	12/12	0/0
Kumar (2019)	India	30–45	NA	NA	NA	NA	7/7	0/0	
Khormizi and Azarniveh (2017)	Iran	51.06 ± 2.3	51.2 ± 3.7	73.77 ± 9.78	74.61 ± 11.57	30.04 ± 0.9	31.73 ± 1.02	15/15	0/0
Batar et al. (2020)	Turkey	41.80 ± 3.21	41.10 ± 2.80	NA	NA	29.24 ± 2.24	30.21 ± 2.14	30/30	0/0
Zhang and Chen (2019)	China	67.58 ± 3.02	67.52 ± 3.06	NA	NA	29.84 ± 0.93	29.88 ± 0.91	28/28	0/0
Cui et al. (2020)	China	68.04 ± 4.78	69.50 ± 4.25	NA	NA	21.85 ± 3.02	22.39 ± 3.10	44/46	0/0

**Table:** 

**References**	**Population group**	**Study design**	**Intervention**	**Intervention dose**	**Main outcome**
Marinda et al. (2013)	Elderly women	RCT	PG: Mat Pilates CG: Non-exercising	180 min/w (60 min, 3 d/w); 8 w	FBG, TC, TG
Aslan et al. (2019)	Obese people	RCT	PG: Pilates+ diet CG: Diet	120 min/w (60 min, 2 d/w); 6 w	FBG
Ramezankhany et al. (2011)	Sedentary women	RCT	PG: Pilates+ diet CG: Diet	135 min/w (45 min, 3 d/w); 16 w	TC, HDL-C, LDL-C, TG, HDL-C/LDL-C
Melo et al. (2020)	Older women with type 2 diabetes	RCT	PG: Pilates+ medications and dietary treatments CG: Medications and dietary treatments	180 min/w (60 min, 3 d/w); 12 w	FBG, PBG, HbA1c
Yucel and Uysal (2018)	Women with type 2 Diabetes	RCT	PG: Pilates+ medications and dietary treatments CG: Medications and dietary treatments	180–210 min/w (45–70 min, 3 d/w); 12 w	FBG, PBG, HbA1c
Jung et al. (2020)	Women with obesity	RCT	PG: Pilates CG: Non-exercising	150 min/w (50 min, 3 d/w); 12 w	TC, HDL-C, LDL-C, TG, FBG, Insulin, HOMA-IR, HOMA-β
Tunar et al. (2012)	Adolescents with type 1 diabetes	RCT	PG: Pilates+ medications CG: Medications	135 min/w (45 min, 3 d/w); 12 w	Daily insulin dose, TC, HDL-C, LDL-C, TG, HbA1c
Abd EL-Monim et al. (2019)	Obese women with type 2 diabetes	RCT	PG: Pilates+ medications CG: Medications	180 min/w (60 min, 3 d/w); 12 w	TC, HDL-C, LDL-C, TG, HbA1 c
Gokgul and Hazar (2017)	Sedentary women	RCT	PG: Pilates CG: Cyclic exercises	90 min/w (30 min, 3 d/w); 8 w	HDL-C, LDL-C
Zolfaghari et al. (2015)	Women with type 2 diabetes	RCT	PG: Pilates CG: Non-exercising	225 min/w (75 min, 3 d/w); 8 w	FBG, HbA1 c
Kumar (2019)	Middle-aged women	RCT	PG: Pilates CG: Brisk walking	3 d/w; 12 w	FBG
Khormizi and Azarniveh (2017)	Obese women with type 2 diabetes	RCT	PG: Pilates CG: Non-exercising	180 min/w (60 min, 3 d/w); 8 w	FBG, HbA1 c
Batar et al. (2020)	Women with type 2 diabetes	RCT	PG: Pilates+ medications and dietary treatments CG: Medications and dietary treatments	180 min/w (60 min, 3 d/w); 12 w	TC, HDL-C, LDL-C, TG, Insulin, FBG, HbA1c
Zhang and Chen (2019)	Patients with type 2 diabetes	RCT	PG: Pilates+ medications and dietary treatments CG: Medications and dietary treatments	420 min/w (30 min, twice a day), 8 w	TC, LDL-C, TG, FBG, PBG, HbA1c
Cui et al. (2020)	Patients with type 2 diabetes	RCT	PG: Pilates+ medications and dietary treatments CG: Medications and dietary treatments	210 min/w (30 min, once a day), 12 w	FBG, PBG, HbA1c

*FBG, fasting blood glucose; PBG, post-prandial blood glucose; HbA1c, glycated hemoglobin; HOMA-IR, homeostatic model assessment for insulin resistance; HOMA-β, homeostatic model assessment for beta cells; TC, total cholesterol; TG, triglycerides; HDL-C, high density lipoprotein cholesterol; LDL-C, low-density lipoprotein cholesterol*.

### Risk of Bias and Quality Assessment

**Figure 2:**
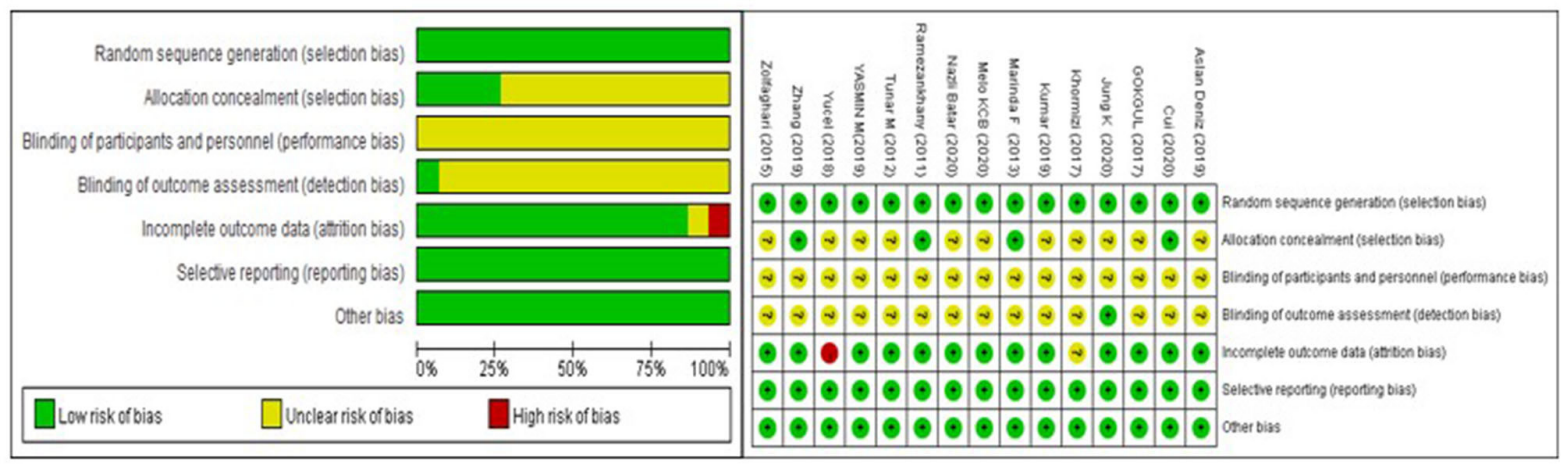
Risk of bias graph.

The risk-of-bias assessment is shown in Figure 2. All studies were described as randomly generated, while only five of them (Ramezankhany et al., 2011; Marinda et al., 2013; Zhang and Chen, 2019; Batar et al., 2020; Cui et al., 2020) recorded the methods of randomization in detail. Blinding was described in one study (Jung et al., 2020). The drop-out rate was reported in three articles (Gokgul and Hazar, 2017; Yucel and Uysal, 2018; Jung et al., 2020), one (Yucel and Uysal, 2018) of which was defined as a high risk of bias because its drop-out rate was more than 15%.

### Effects on Glucose Metabolism

#### Fasting Blood Glucose

**Figure 3:**
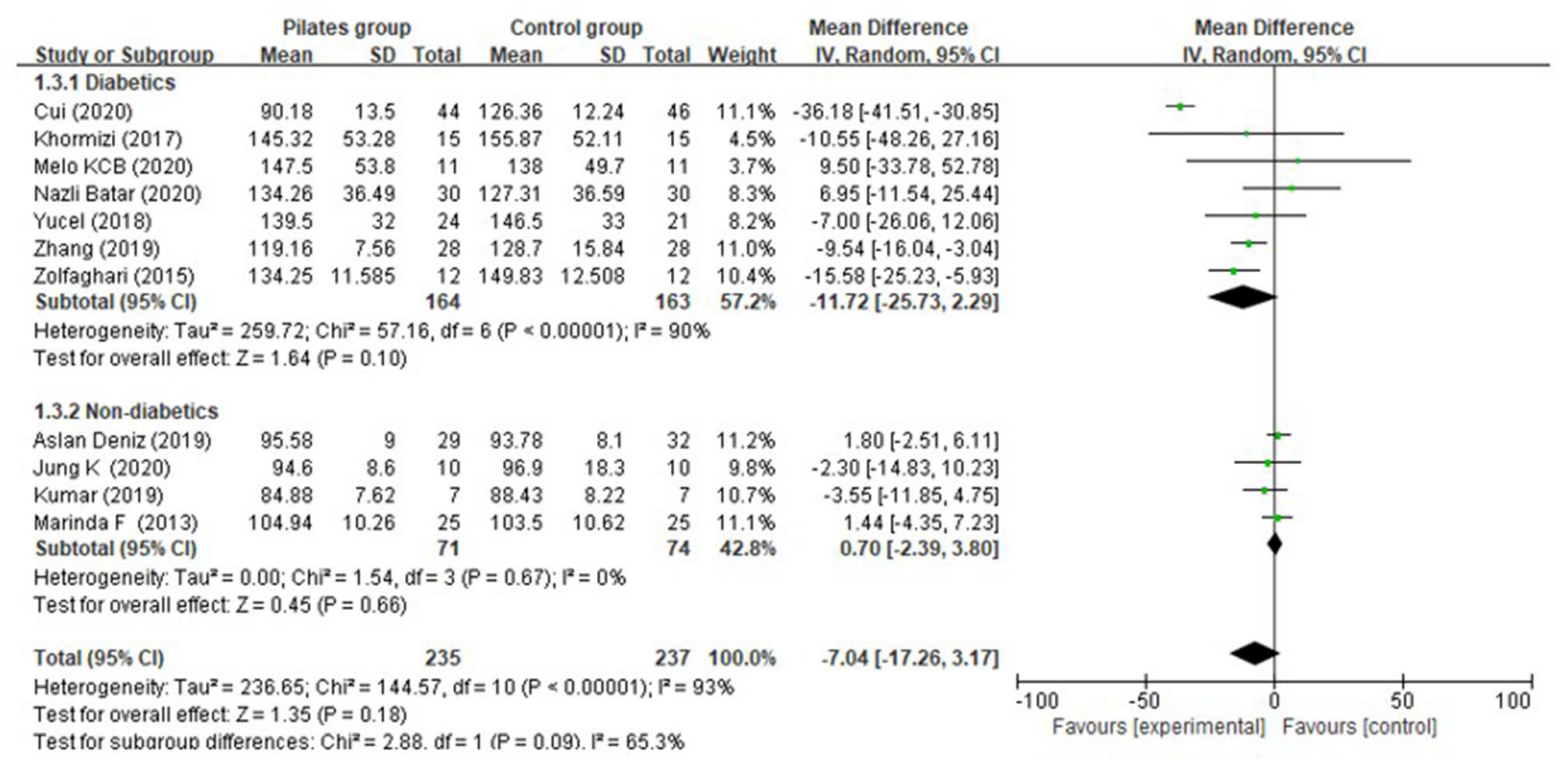
Meta-analysis and forest plot for fasting blood glucose.

Eleven (Marinda et al., 2013; Zolfaghari et al., 2015; Khormizi and Azarniveh, 2017; Yucel and Uysal, 2018; Aslan et al., 2019; Kumar, 2019; Zhang and Chen, 2019; Batar et al., 2020; Cui et al., 2020; Jung et al., 2020; Melo et al., 2020) of the included studies investigated the effect of Pilates on fasting blood glucose, of which seven assessed people suffering from diabetes, and the other four assessed non-diabetic persons. From the random-effects model, overall, no significant difference was detected between Pilates group (PG) and control group (CG) in fasting blood glucose (MD = −7.04 mg/dL, 95% CI: [−17.26, 3.17] mg/dL, *P* = 0.18, I^2^ = 93%). After subgroup analysis, we found that there was no significant difference in fasting blood glucose between the PG and CG, either for diabetic patients (MD = −11.72 mg/dL, 95% CI: [−25.73, 2.29] mg/dL, *P* = 0.10, I^2^ = 90%) or non-diabetic subjects (MD = 0.70 mg/dL, 95% CI: [−2.39, 3.80] mg/dL, *P* = 0.66, I^2^ = 0%) (Figure 3). We also evaluated the effect of Pilates on fasting blood glucose with different intervention durations. However, the reductions in fasting blood glucose were closely similar between the PG and CG after 8-week (MD = −4.84 mg/dL, 95% CI: [−11.92, 2.24] mg/dL, *P* = 0.18, I^2^ = 77%) and 12-week Pilates training (MD = −7.33 mg/dL, 95% CI: [−25.74, 11.07] mg/dL, *P* = 0.43, I^2^ = 93%) (Supplementary Figure 3). Further analysis of the diabetic population indicated that PG revealed a more significant improvement than CG in fasting blood glucose after 8-week intervention (MD = −11.41 mg/dL, 95% CI: [−16.75, −6.07] mg/dL, *P* < 0.0001, I^2^ = 0%), while PG had no significant difference in lowering fasting blood glucose (MD = −9.26 mg/dL, 95% CI: [−36.09, 17.56] mg/dL, *P* = 0.50, I^2^ = 90%) when compared to CG after 8 weeks of intervention (Supplementary Figure 4). Meanwhile, PG showed no significant decrease in fasting blood glucose for non-diabetic subjects after 8 weeks of Pilates exercise (MD = −3.17 mg/dL, 95% CI: [−10.09, 3.75] mg/dL, *P* = 0.37, I^2^ = 0%) and 12 weeks (MD = 1.67 mg/dL, 95% CI: [−1.79, 5.13] mg/dL, *P* = 0.34, I^2^ = 0%) compared to the CG. Most importantly, subgroup analysis by the different comparison of intervention between the PG and CG among diabetic patients indicated that Pilates was superior to non-exercise in decreasing fasting blood glucose (MD = −15.27 mg/dL, 95% CI: [−24.62, −5.93] mg/dL, *P* = 0.001, I^2^ = 0%), whereas no difference was detected in the two comparisons: Pilates + medications vs. medications, only one study (Yucel and Uysal, 2018) reported no significant difference in fasting blood glucose between the two groups; Pilates + medications and dietary treatments vs. medications and dietary treatments: (MD = −10.90 mg/dL, 95% CI: [−32.35, 10.54] mg/dL, *P* = 0.32, I^2^ = 94%) (Supplementary Figure 5).

#### Post-prandial Blood Glucose

**Figure 4:**
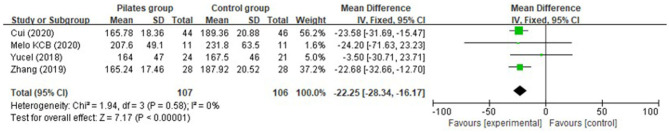
Meta-analysis and forest plot for post-prandial blood glucose.

Four of the included studies (Yucel and Uysal, 2018; Zhang and Chen, 2019; Cui et al., 2020; Melo et al., 2020) reported post-prandial blood glucose, of which the diabetic patients received Pilates combined with medications and dietary treatments in the PG, and a combination of medications and dietary treatments were used in the CG. From the fixed effect model, we found that PG presented with a significantly lower post-prandial blood glucose than the CG (MD = −22.25 mg/dL, 95% CI: [−28.34, 16.17] mg/dL, *P* < 0.00001, I^2^ = 0%), and the heterogeneity was small (Figure 4). Moreover, a significantly greater effect could be observed in the PG than that in the CG both after 8-week intervention [only one study reported (Zhang and Chen, 2019), MD = −22.68 mg/dL, 95% CI: [−32.66, −12.70] mg/dL, *P* < 0.00001, I^2^ was not applicable] and 12-week intervention (MD = −22.00 mg/dL, 95% CI: [−29.67, −14.33] mg/dL, *P* < 0.00001, I^2^ = 0%).

#### Insulin

**Figure 5:**
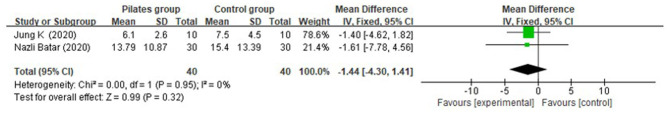
Meta-analysis and forest plot for insulin.

With respect to insulin concentration, no significant difference was found between the PG and CG (MD = −1.44 μU/mL, 95% CI: [−4.30, 1.41] μU/mL, *P* = 0.32, I^2^ = 0%) (Figure 5).

#### Glycated Hemoglobin (HbA1c)

**Figure 6:**
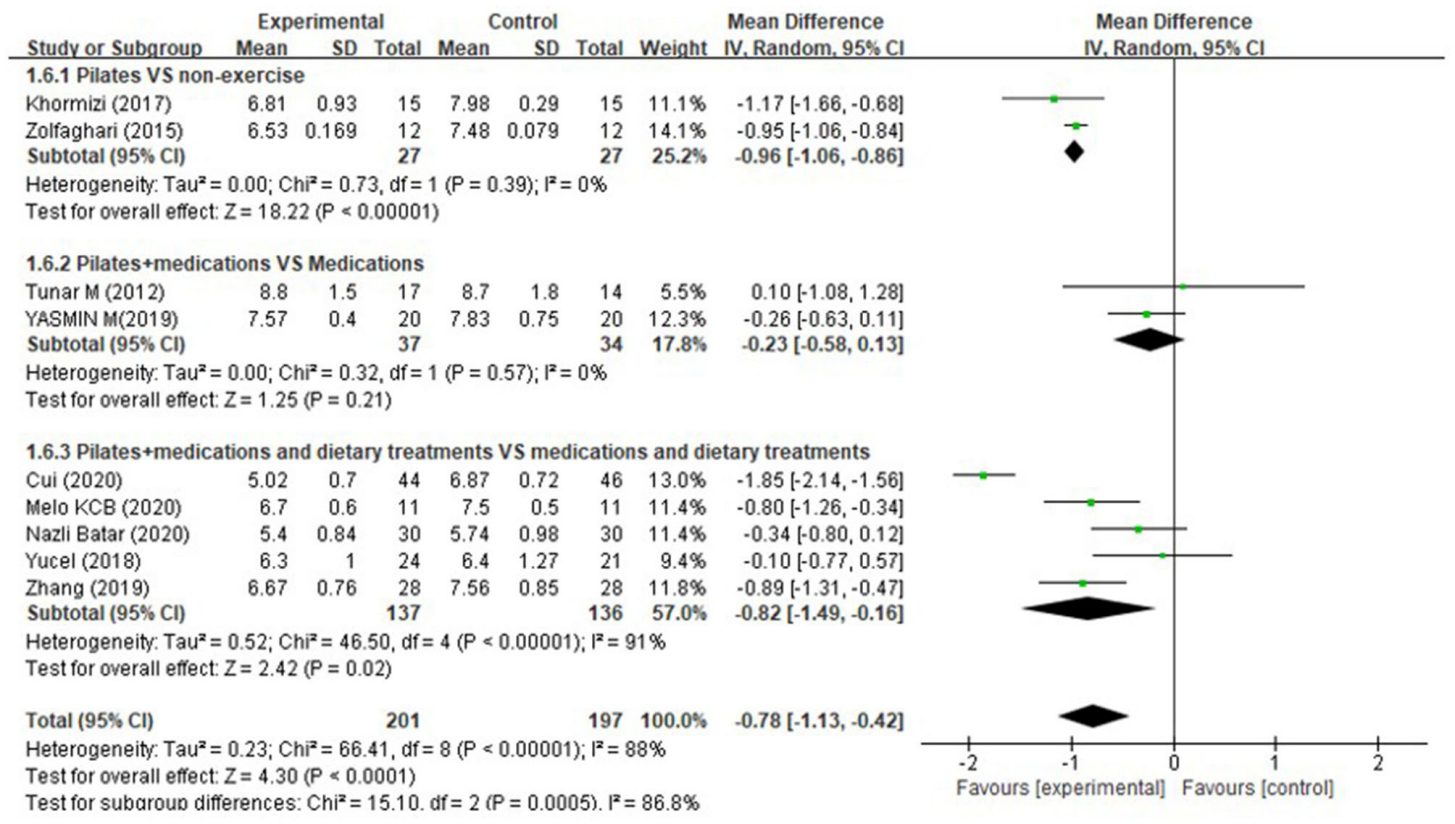
Meta-analysis and forest plot for glycated hemoglobin (HbA1c).

Among diabetic patients from nine studies (Tunar et al., 2012; Zolfaghari et al., 2015; Khormizi and Azarniveh, 2017; Yucel and Uysal, 2018; Abd EL-Monim et al., 2019; Zhang and Chen, 2019; Batar et al., 2020; Cui et al., 2020; Melo et al., 2020), a significant overall effect on glycated hemoglobin (HbA1c) could be seen for Pilates, but the heterogeneity was high (MD = −0.78%, 95% CI: [−1.13, −0.42]%, *P* < 0.0001, I^2^ = 88%). Of note, subgroup analysis suggested that Pilates showed a significant reduction in lowering HbA1c when compared to non-exercising (MD = −0.96%, 95% CI: [−1.06, −0.86]%, *P* < 0.00001, I^2^ = 0%). Meanwhile, Pilates combined with medications and dietary treatments presented a greater reduction in HbA1c than a combination of medications and dietary treatments (MD = −0.82%, 95% CI: [−1.49, −0.16]%, *P* < 0.0001, I^2^ = 88%). However, Pilates, as an adjunctive treatment to medications was not superior to medications used alone (MD = −0.23%, 95% CI: [−0.58, 0.13]%, *P* = 0.21, I^2^ = 0%), (Figure 6). Diabetic patients from the three studies (Zolfaghari et al., 2015; Khormizi and Azarniveh, 2017; Zhang and Chen, 2019) receiving 8-week interventions, and the HbA1c was pronouncedly higher in the PG than that in the CG after intervention (MD = −0.96%, 95% CI: [−1.06, −0.86]%, *P* < 0.0001, I^2^ = 0%). Six studies (Tunar et al., 2012; Yucel and Uysal, 2018; Abd EL-Monim et al., 2019; Batar et al., 2020; Cui et al., 2020; Melo et al., 2020) reported a 12-week intervention, and we found that diabetes patients receiving 12 weeks of Pilates training showed a greater reduction in HbA1c than those with control conditions (MD = −0.59%, 95% CI: [−1.29, −0.11]%, *P* = 0.10, I^2^ = 92%) (Supplementary Figure 6).

### Effects on Lipids Metabolism

#### Total Cholesterol

**Figure 7:**
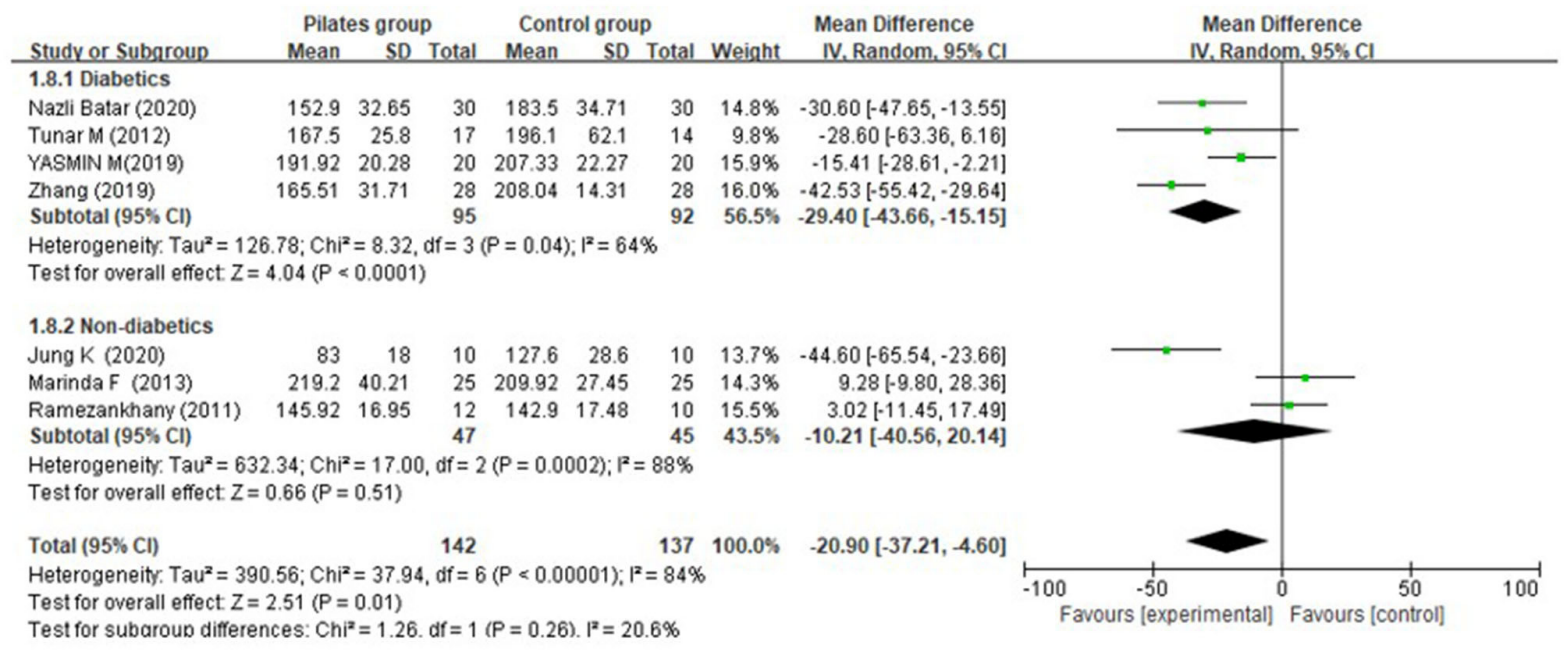
Meta-analysis and forest plot for total cholesterol.

Seven (Ramezankhany et al., 2011; Tunar et al., 2012; Abd EL-Monim et al., 2019; Aslan et al., 2019; Zhang and Chen, 2019; Batar et al., 2020; Jung et al., 2020) among the included studies tested total cholesterol, of which three studies (Ramezankhany et al., 2011; Aslan et al., 2019; Jung et al., 2020) documented it for diabetic patients and four studies (Tunar et al., 2012; Abd EL-Monim et al., 2019; Batar et al., 2020; Cui et al., 2020) for non-diabetic subjects. Overall, Pilates appeared to lower total cholesterol (MD = −20.90 mg/dL, 95% CI: [−37.21, −4.60] mg/dL, *P* = 0.01, I^2^ = 84%). Compared to the CG, PG presented a significant reduction in total cholesterol for individuals affected by diabetes (MD = −29.40 mg/dL, 95% CI: [−43.66, −15.15] mg/dL, *P* < 0.0001, I^2^ = 64%), both at post-intervention week 8 [only one study (Zhang and Chen, 2019) reported, MD = −42.53 mg/dL, 95% CI: [−55.42, −29.64] mg/dL, *P* < 0.0001, I^2^ was not applicable] and 12 (MD = −21.84 mg/dL, 95% CI: [−32.12, −11.56] mg/dL, *P* < 0.0001, I^2^ = 3%), whereas a closely similar effect on total cholesterol was found in non-diabetic persons (MD = −10.21 mg/dL, 95% CI: [−40.56, 20.14] mg/dL, *P* = 0.51, I^2^ = 88%) (Figure 7).

#### Triglycerides

**Figure 8:**
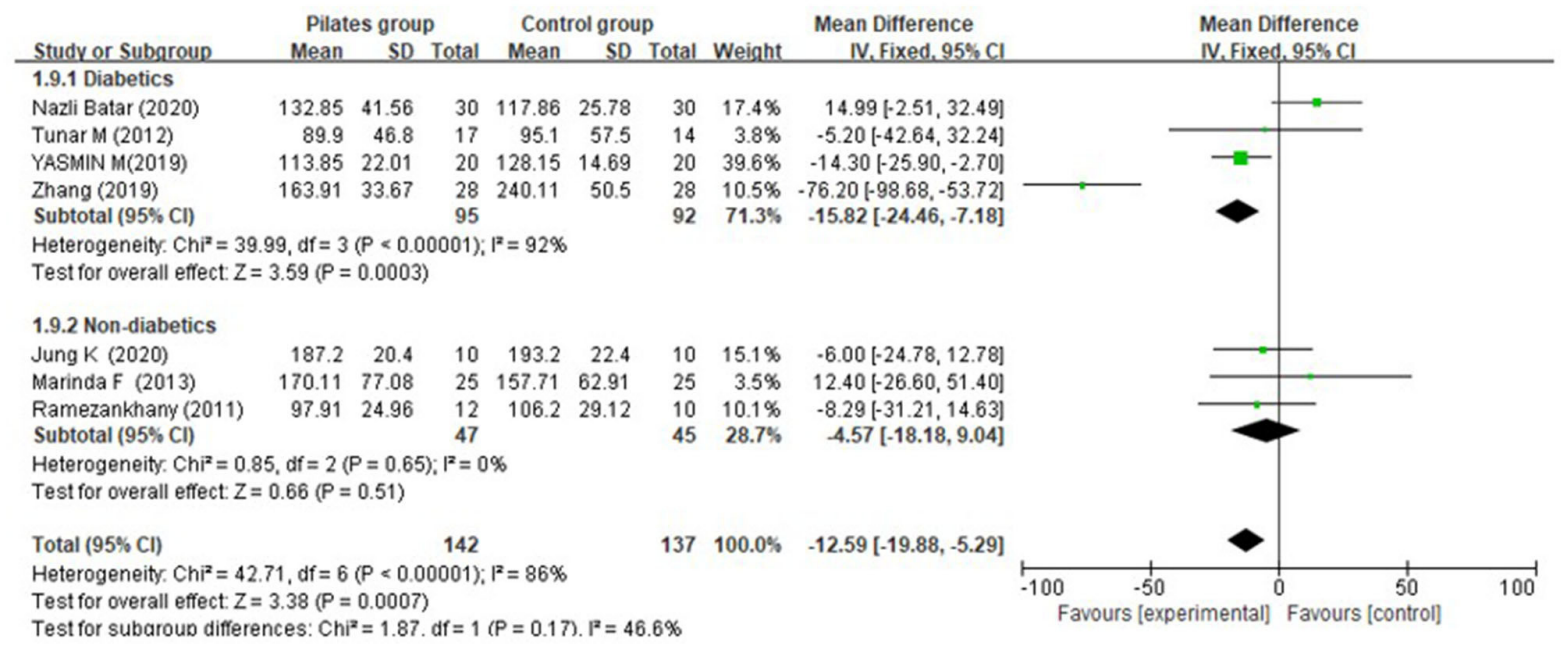
Meta-analysis and forest plot for triglycerides.

Seven studies (Ramezankhany et al., 2011; Tunar et al., 2012; Abd EL-Monim et al., 2019; Aslan et al., 2019; Zhang and Chen, 2019; Batar et al., 2020; Jung et al., 2020) reported triglycerides, of which three studies (Ramezankhany et al., 2011; Aslan et al., 2019; Jung et al., 2020) described it for diabetic patients and four studies (Tunar et al., 2012; Abd EL-Monim et al., 2019; Zhang and Chen, 2019; Batar et al., 2020) for non-diabetic subjects. Overall, a significant effect was determined (MD = −12.59 mg/dL, 95% CI: [−19.88, −5.29] mg/dL, *P* = 0.0007, I^2^ = 86%). Subgroup analyses by population group demonstrated that there was a significant difference between the PG and CG for subjects with diabetes (MD = −15.82 mg/dL, 95% CI: [−24.46, −7.18] mg/dL, *P* = 0.0003, I^2^ = 92%), and the effect sizes of 8 and 12-week interventions were pooled from one study (Zhang and Chen, 2019) (MD = −76.20 mg/dL, 95% CI: [−98.68, −53.72] mg/dL, *P* < 0.0001, I^2^ was not applicable) and three studies (Tunar et al., 2012; Abd EL-Monim et al., 2019; Batar et al., 2020) (MD = −5.53 mg/dL, 95% CI: [−14.71, 4.01] mg/dL, *P* = 0.26, I^2^ = 73%) respectively, while both groups showed a similar effect for non-diabetic persons (MD = −4.57 mg/dL, 95% CI: [−18.18, 9.04] mg/dL, *P* = 0.51, I^2^ = 0%) (Figure 8).

#### High Density Lipoprotein Cholesterol (HDL-C)

**Figure 9:**
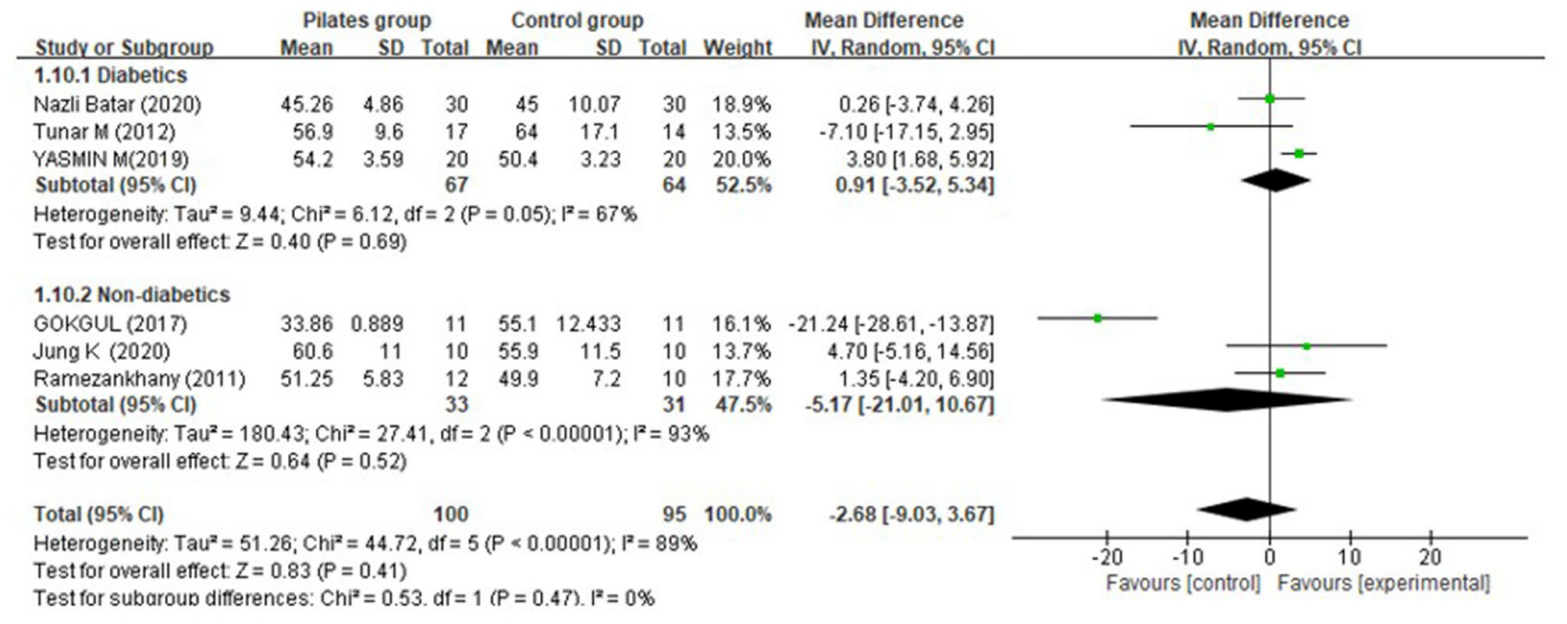
Meta-analysis and forest plot for high-density lipoprotein cholesterol.

Overall, with respect to HDL-C, the PG showed no significantly greater increase in contrast to the CG (MD = −2.68 mg/dL, 95% CI: [−9.03, 3.67] mg/dL, *P* = 0.41, I^2^ = 89%). Furthermore, similar and non-significant effect sizes were found both in diabetes (MD = 0.91 mg/dL, 95% CI: [−3.52, 9.94] mg/dL, *P* = 0.69, I^2^ = 67%) after a 12-week intervention, and non-diabetic individuals (MD = −5.17 mg/dL, 95% CI: [−21.01, 10.67] mg/dL, *P* = 0.52, I^2^ = 93%) (Figure 9).

#### Low Density Lipoprotein Cholesterol (LDL-C)

**Figure 10:**
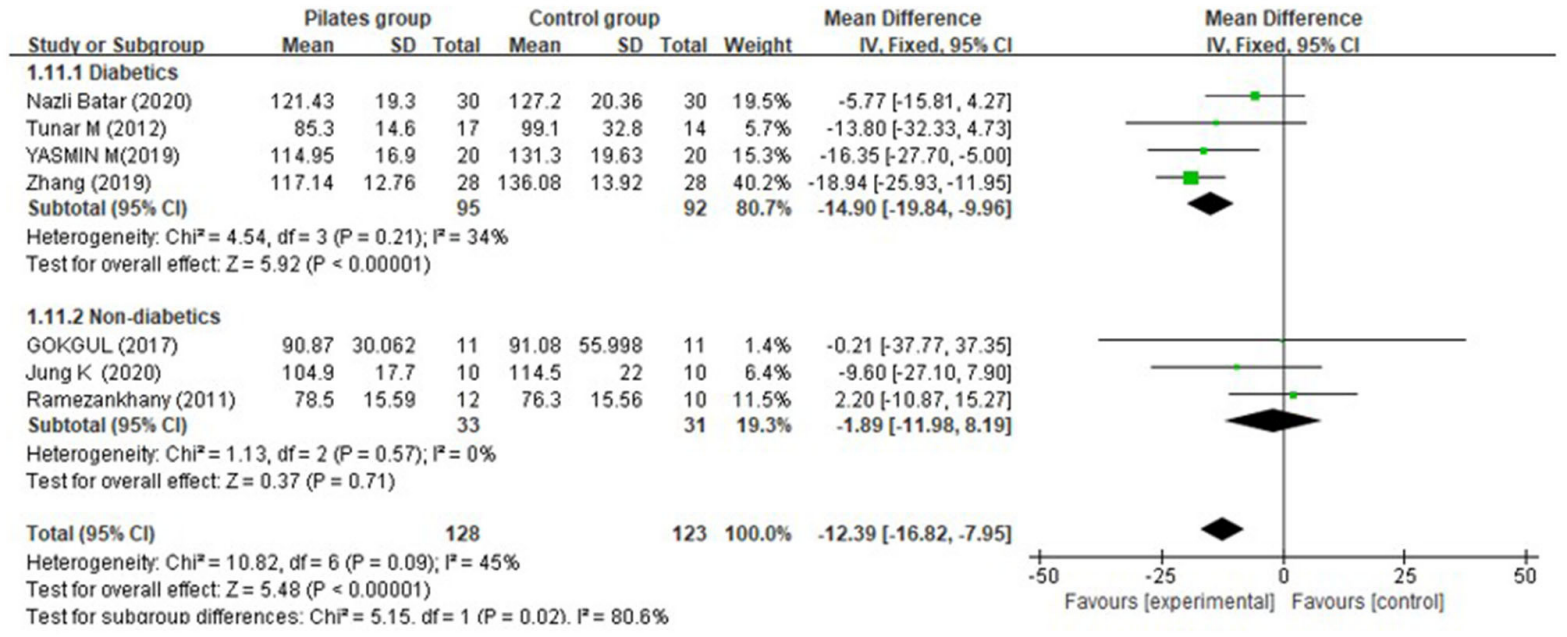
Meta-analysis and forest plot for low-density lipoprotein cholesterol.

Meta-analysis of five trials found that the overall effect was significant (MD = −12.39 mg/dL, 95% CI: [−16.82, −7.95] mg/dL, *P* < 0.00001, I^2^ = 45%). Subgroup analysis indicated that there was a greater decrease of LDL-C in the PG compared to the CG for diabetic patients (MD = −14.90 mg/dL, 95% CI: [−19.84, −9.96] mg/dL, *P* < 0.00001, I^2^ = 34%) at post-intervention week 12, but no significant difference was detected for non-diabetic participants (MD = −1.89 mg/dL, 95% CI: [−11.98, 8.19] mg/dL, *P* = 0.71, I^2^ = 0%) (Figure 10).

### Subgroup Analysis by Intensity of Pilates

From 10 (Ramezankhany et al., 2011; Tunar et al., 2012; Marinda et al., 2013; Khormizi and Azarniveh, 2017; Yucel and Uysal, 2018; Abd EL-Monim et al., 2019; Aslan et al., 2019; Batar et al., 2020; Jung et al., 2020; Melo et al., 2020) of the 15 included studies, patients received Pilates training from 120 min/w to 180 min/w; there were 3 other studies (Zolfaghari et al., 2015; Zhang and Chen, 2019; Cui et al., 2020) reporting Pilates exercise intensity more than 200 min/w. We found that when diabetic patients received Pilates more than 200 min/w, they had a greater improvement in fasting blood glucose (MD = −20.59 mg/dL, 95% CI: [−38.88, −2.30] mg/dL, *P* = 0.03, I^2^ = 95%), and post-prandial blood glucose (MD = −23.22 mg/dL, 95% CI: [−29.52, −16.93] mg/dL, *P* < 0.0001, I^2^ = 0%) compared to patients on the waiting list. However, no significant difference was detected between the two groups in fasting blood glucose (MD = −0.19 mg/dL, 95% CI: [−12.21, 11.84] mg/dL, *P* = 0.98, I^2^ = 0%) and post-prandial blood glucose (MD = −8.63 mg/dL, 95% CI: [−32.23, 14.98] mg/dL, *P* = 0.4, I^2^ = 0%) when the intensity of Pilates exercise was <200 min/w. With respect to HAb1c, we found that the minimum response intensity of Pilates exercise was 120 min/w. For non-diabetes subjects, only one study (Gokgul and Hazar, 2017) reported a Pilates intensity of 90 min/w, and there was still no difference between both groups in HDL-C and LDL-C when the study was eliminated.

### Sensitivity and Subgroup Analyses

The pooled assessment on HbA1c presented with high heterogeneity, so we conducted a sensitivity analysis for it. Through removing studies from the analyses individually, we found that the overall heterogeneities and results had no substantial change (Table 2).

**Table 2:** Sensitivity analysis for HbA1c.

**References**	**Effect size**	**95% CI**	***P***	**I^**2**^**
Cui et al. (2020)	−0.64	−0.92, −0.36	*P < *0.00001	74%
Khormizi and Azarniveh (2017)	−0.72	−1.11, −0.33	*P =* 0.0003	89%
Melo et al. (2020)	−0.77	−1.16, −0.37	*P =* 0.0001	89%
Batar et al. (2020)	−0.83	−1.21, −0.46	*P < *0.0001	88%
Tunar et al. (2012)	−0.83	−1.19, −0.47	*P < *0.00001	89%
Abd EL-Monim et al. (2019)	−0.85	−1.22, −0.49	*P < *0.00001	87%
Yucel and Uysal (2018)	−0.85	−1.21, −0.48	*P < *0.00001	88%
Zhang and Chen (2019)	−0.75	−1.15, −0.35	*P =* 0.0002	89%
Zolfaghari et al. (2015)	−0.72	−1.23, −0.21	*P =* 0.006	89%

*CI, confidence intervals*.

### Publication Bias

As is summarized in Table 3, there was no evidence for significant publication bias evaluated by using Egger's regression and Begg's tests.

**Table 3:** Assessment of publication bias.

**Outcomes**	***N***	**Begg' s test**	**Egger's test**
FBG (8w)	4	0.221	0.273
FBG (12w)	4	0.452	0.828
FBG (merged)	8	0.062	0.775
PBG	2	0.308	0.487
Insulin	2	1	NA
HbA1c	6	0.348	0.154
TC	5	1	0.685
TG	5	0.548	0.554
HDL-C	5	0.707	0.362
LDL-C	5	0.230	0.416

*FBG, fasting blood glucose; PBG, post-prandial blood glucose; HbA1c, glycated hemoglobin; HDL-C, high density lipoprotein cholesterol; LDL-C, low-density lipoprotein cholesterol; TC, total cholesterol; TG, triglycerides; NA, not available*.

## Discussion

Exercise is considered as a therapeutic cornerstone among patients with metabolic diseases (Sylow et al., 2017). It can enhance insulin sensitivity, and promote glucose transport and metabolism (Borghouts and Keizer, 2000), which has great benefits for glucose control. During the training program, exercise requires the expenditure of energy deriving from the supply of blood glucose, and consequently decreases the blood glucose level. Meanwhile, regular exercise can significantly improve blood lipid levels. It is proven that exercise exerts positive impacts on the pathogenesis of subjects with dyslipidemia, and reduces cholesterol levels (Mann et al., 2014). As reported, mind-body exercise has been shown to be beneficial to lower triglyceride and fasting glucose levels (Younge et al., 2015). Pilates, as one mind-body exercise, was reported to have positive effects on cardiorespiratory fitness (Hagner-Derengowska et al., 2015) and sleep quality (Chen et al., 2020), which is closely related to the metabolism of the body. Recently, Pilates has attracted a lot of attention and been increasingly used to improve glucose and lipids metabolism in clinic practice. However, whether it is worth being explicitly recommended still lacks evidence.

To the best of our knowledge, this is the first systematic review and meta-analysis to examine the effects of Pilates exercise on blood glucose and lipids metabolism. In the present study, we found that Pilates exercise could significantly lower fasting blood glucose and HbA1c among diabetic patients when compared to non-exercising, while it seemed to be ineffective in decreasing fasting blood glucose for non-diabetic individuals. Diabetic patients live with impaired glucose metabolism showing hyperglycemia, while participants without diabetes always present a normal glycemia with low variability. It could be the reason that the effects of Pilates on fasting blood glucose were not significant among non-diabetic subjects. The previous meta-analyses (Umpierre et al., 2011; Sampath Kumar et al., 2019) highlighted that regular exercise could be an effective interventional strategy to improve fasting blood glucose and HbA1c for diabetic patients, which was in accordance with our results. HbA1c was a biomarker for diagnosing diabetes mellitus, and the gold standard reflecting glycemic control (Khan et al., 2020), which played a critical role in the treatment of diabetes. However, subgroup analysis of HbA1c and fasting blood glucose indicated that Pilates, as a supplementary treatment to medications, was not superior to the treatments used alone in diabetic patients. For the treatment of diabetes, pharmacological methods still served as the first-line therapies, to achieve an excellent blood glucose management. Thus, the effects of Pilates on fasting blood glucose and HbA1c were unobvious when patients received pharmacological treatments. Meanwhile, our finding revealed that Pilates combined with medications and dietary treatments showed no significant improvement in reducing fasting blood glucose, whereas it had a greater reduction in post-prandial blood glucose and HbA1c for diabetic patients. It could be explained by the reason that dietary treatment exerted a key impact on post-prandial blood glucose, as high sugar diet could cause higher post-prandial blood glucose, and long-term unsatisfactory blood glucose management would lead to abnormal glycated hemoglobin (HbA1c). In addition, Pilates showed no effect on insulin concentration in the blood compared to the control condition. Therefore, even though a significantly greater decrease of post-prandial blood glucose was observed in the PG than that in the CG after more than 8 weeks of intervention, the efficacy of Pilates on post-prandial blood glucose was still unconfirmed due to the various factors for it, including intake and diet habits.

Regarding the effects on blood lipids, the results derived from this study revealed that, compared to the control condition, Pilates presented a non-significant effect on blood lipids, including TC, TG, HDL-C, and LDL-C, for non-diabetic individuals. These findings were in agreement with a previous study suggesting that Pilates almost had no efficacy for lipids metabolism in healthy subjects (Kim et al., 2014). In the previous study, it was reported that body fat ratio was a factor positively affecting lipid metabolism (Kondo et al., 2006). While, the latest meta-analysis suggested that Pilates showed no significantly greater reduction of body composition than other interventions (Cavina et al., 2020). Therefore, it could be the reason that Pilates had no effect on lipids reduction for non-diabetic individuals. Nevertheless, we found that Pilates had a significant improvement in lowering TC, TG, and LDL-C, except for HDL-C in patients with diabetes. In the previous meta-analysis, aerobic exercise was found to be effective to lower LDL-C in adults with type 2 diabetes. In our study, Pilates exercise also had this efficacy, which might be explained by the larger variability of lipids and its possible influence on the cross-talk of hyperglycemia and dyslipidemia in diabetes, which consequently decreases blood lipids. In addition, as was reported, an increase in TG and LDL-C concentrations was a direct cause of cardiovascular disease (Wallace et al., 1997). It might be the reason why Pilates was recommended during the prevention and treatment of cardiovascular disease.

From the subgroup analysis of intervention dose for diabetic patients, we found that both 8-week and 12-week Pilates exercise could decrease levels of post-prandial blood glucose, TC, and LDL-C. However, regarding fasting blood glucose, HbA1c, and TG, while the effects of Pilates on reducing their levels could be detected after 8-week intervention, those positive efficacies could not be observed after 12-week intervention. Thus, our findings demonstrated that the benefits of Pilates in lowing fasting blood glucose, HbA1c, and TG were concentrated in the 8-week post-intervention phase but were ineffective in the long run. Exercise could promote the metabolism and transformation of glucose. However, insulin resistance existed in diabetes mellitus, which was detrimental for muscle cells to absorb and store glucose and triglycerides, and contributed to high levels of glucose and triglycerides in the blood (Misra et al., 2008). Because Pilates showed no effect on insulin in the blood, the benefits of Pilates disappear gradually as the body adapts to the consumption of glucose and lipids by Pilates. The results from subgroup analysis by intensity of Pilates indicated that, for diabetic patients, the effects of Pilates on blood glucose could be influenced not only by the duration of the intervention but also by its intensity. Moreover, it seemed that its intensity rather than duration was more important for diabetics.

Pilates, as a mind-body exercise, aims to enhance core stability, strength, and flexibility, through exercises with more control of movement (de Oliveira et al., 2019). The findings in this study were in accordance with two recently published meta-analyses for another similar exercise, yoga, which suggesting that it could reduce TC and LDL-C significantly, but had no significant effect on TG and HDL-C (Azami et al., 2019), and it could improve HbA1c and post-prandial blood glucose (Thind et al., 2017). However, the effects of yoga frequency and intervention doses on glucose and lipids were still unreported. In this study, we found that the effects of Pilates were varied with population groups, intervention doses, and even combination treatment types, which should be the reason contributing to inconsistent results among different studies. According to the previous studies, a greater favorable result was observed in patients with chronic lower back pain receiving Pilates than those treated with home exercise (Batibay et al., 2020).

Moreover, Pilates was considered be effective to improve mood disorders for overweight/obese individuals (Vancini et al., 2017). In addition, Pilates exercise mixes yoga, gymnastics, martial arts, and dance with philosophical notions. It was suggested that Pilates was more effective for improving functional movement and individual health level to assess quality of life than yoga and the control condition (Lim and Park, 2019; Abasiyanik et al., 2020). But Dunleavy et al. (2016) reported that no favorable results were found in Pilates compared to yoga. Thus, Pilates might be superior to yoga or other exercise for the treatment of certain diseases. Most importantly, Pilates had fewer side effects on joints than weight baring exercise, such as jogging and running, especially for overweight/obese people, or those with joint problems. However, whether Pilates is a better strategy to lower blood glucose and lipids is unreported until now.

Some limitations must be considered in our study. First, random and blind methods of the included studies were rarely recorded in detail. Second, non-diabetic participants of the included studies comprised sedentary, obese, middle-aged, and elderly people, which could affect the results due to their different blood glucose and lipids status. Third, most of the subjects in the included studies was female, while gender could be a factor influencing glucose and lipids metabolism. Fourth, Pilates types and procedure processes varied from one study to another without a consolidated standard, which could be potential sources that affected the results.

Future research should still concentrate on the effect of Pilates on blood glucose and lipids metabolism, especially for non-diabetic people. In order to eliminate the heterogeneity as far as possible, some conditions including age, gender, sedentary, and obese should be taken into consideration to draw comparisons. Most importantly, well-designed and large-scale RCTs comparing the effects of Pilates combined with medications vs. medications alone are required to confirm whether Pilates had a synergism with medications in glucose and lipids metabolism for diabetic patients.

## Conclusion

In this analysis, we systematically reviewed and quantified the effects of Pilates on blood glucose and lipids. Overall, compared to non-exercising or control conditions, Pilates had no significant effect on glucose and lipids metabolism for non-diabetic individuals, whereas it could significantly improve blood glucose and lipids metabolism for diabetic patients, including the reduction of post-prandial blood glucose, fasting blood glucose, HbA1c, TG, TC, and LDL-C, but the effects on blood glucose in diabetics could be influenced not only by the duration of the intervention but also by its intensity. Furthermore, for diabetic patients, Pilates combined with medications showed no significantly greater reduction in fasting blood glucose than medications used alone, and Pilates combined with medications and dietary treatments showed no significant improvement in reducing fasting blood glucose, whereas it had a greater reduction in post-prandial blood glucose and HbA1c. Given the limitation in this work, additional well-designed and large-scale RCTs and systemic reviews are needed to confirm these findings in the future.

## Data Availability Statement

The data analyzed in this study is subject to the following licenses/restrictions: The original data analyzed in this study are presented in the article, further inquiries should be directed to the corresponding authors. Requests to access these datasets should be directed to Zehua Chen, 630327511@qq.com.

## Author Contributions

ZC and XX designed the study. ZC and ZS performed did the literature searches and designed the data-extraction form. ZC and YG selected the studies. ZC and YW extracted the data. ZS and ZC did the statistical analyses. YX, TJ, and HW revised the manuscript. HS, WC, and XY edited the language. XX supervised the study. All authors read and approved the submitted version.

## Conflict of Interest

The authors declare that the research was conducted in the absence of any commercial or financial relationships that could be construed as a potential conflict of interest.
