# Supplementary material for: Effect of Pilates on Glucose and Lipids: A Systematic Review and Meta-Analysis of Randomized Controlled Trials
Source: Front Physiol. 2021 May 28;12:641968. doi: 10.3389/fphys.2021.641968 (PMC8202501; doi:10.3389/fphys.2021.641968)
Supplement: Supplementary file 1 [file Data_Sheet_1.docx]

**Pubmed**

**(randomized controlled trial [Publication Type] OR randomized [Title/Abstract] OR placebo [Title/Abstract]) AND ((****"Exercise Movement Techniques"[Mesh]) OR (((Pilates-Based Exercises) OR (Exercises, Pilates-Based) OR (Pilates Based Exercises) OR (Pilates Training) OR (Training, Pilates)))) 2721**

**Embase**

| No. | Query | Results |
| --- | --- | --- |
| #5 | #3 AND #4 | **524** |
| #4 | 'randomized controlled trial'/exp OR 'controlled clinical trial'/exp OR 'randomized':ti,ab OR 'placebo':ti,ab OR 'drug therapy':lnk OR 'randomly':ti,ab OR 'trial':ti,ab OR 'groups':ti,ab | **7577677** |
| #3 | #1 OR #2 | **976** |
| #2 | 'movement techniques, exercise' OR 'exercise movement technics' OR 'pilates-based exercises' OR 'exercises, pilates-based' OR 'pilates based exercises' OR 'pilates training' OR 'training, pilates' | **139** |
| #1 | 'pilates'/exp OR pilates | **975** |

**Cochran**

ID Search Hits

#1 (pilates):ti,ab,kw (Word variations have been searched)  **610**

#2 ((Pilates-Based Exercises) OR (Exercises, Pilates-Based) OR (Pilates Based Exercises) OR (Pilates Training) OR (Training, Pilates)):ti,ab,kw (Word variations have been searched) **355**

#3 #1 OR #2 **610**

**Web of science:**

TS=(Pilates-Based Exercises OR Exercises, Pilates-Based OR Pilates Based Exercises OR Pilates Training OR Training, Pilates OR Pilates) AND TS=(randomized controlled trial OR randomized) **267**

**CNKI:**

主题=普拉提 or 主题= Pilates or ( 题名= 普拉提 or 题名= Pilates) (精确匹配)  **540**

**Supplementary eFigure 1** The search strategy in this review.

**Non-RCTs**

1. Hagner-Derengowska, Magdalena, et al. "Effects of Nordic Walking and Pilates exercise programs on blood glucose and lipid profile in overweight and obese postmenopausal women in an experimental, nonrandomized, open-label, prospective controlled trial." *Menopause*, 2015; 22 (11): 1215-1223.
2. Kim, Hyo-Jin, Jiyeon Kim, and Chang-Sun Kim. "The effects of pilates exercise on lipid metabolism and inflammatory cytokines mRNA expression in female undergraduates." *Journal of exercise nutrition & biochemistry*, 2014; 18 (3): 267.
3. Suna, Gürhan, and Kenan Isildak. Investigation of the Effect of 8-Week Reformer Pilates Exercise on Flexibility, Heart Rate and Glucose Levels in Sedentary Women. *Asian Journal of Education and Training*, 2020; 6 (2): 226-230.
4. Zandi, Sara, Saeedeh Shadmehri, and Nasibe Kazemi. "The Effect of Pilates Training on Isoprostane, Fasting Glucose and Body Composition in Women with Breast Cancer." *Report of Health Care,* 2016; 2 (4): 26-33
5. Hagner-Derengowska, Magdalena, Krystian Kałużny, and Jacek Budzyński. "Effects of Nordic Walking and Pilates training programs on aminotransferase activity in overweight and obese elderly women." *Journal of Education, Health and Sport,* 2015; 5 (12): 563-580.

**Without full-text**

1. Salem, Leyla, Bahram Abedi, and Mojtaba Khansooz. "The Effect of Six-weeks Pilates Exercise and Cumin Extract Consumption on Lipid Profile and Insulin Resistance Index in Obese and Overweight Women." *Health and Development Journal*, 2018; 7 (4): 295-304.
2. BUTTELLI, Adriana Cristine Koch, et al. "PILATES TRAINING IMPROVES AEROBIC CAPACITY, BUT NOT LIPID OR LIPOPROTEIN LEVELS IN ELDERLY WOMEN WITH DYSLIPIDEMIA: A CONTROLLED TRIAL." *Journal of Bodywork and Movement Therapies* (2020).
3. Erkal ARSLANOĞLU, Ömer ŞENEL. Effects Of Pilates Training On Some Physiological Parameters And Cardiovascular Risk Factors Of Middle Aged Sedentary Women[J]. Ijssjournal Com, 2013.

**Supplementary eFigure 2** Fifteen excluded studies according to the inclusion and exclusion criteria.


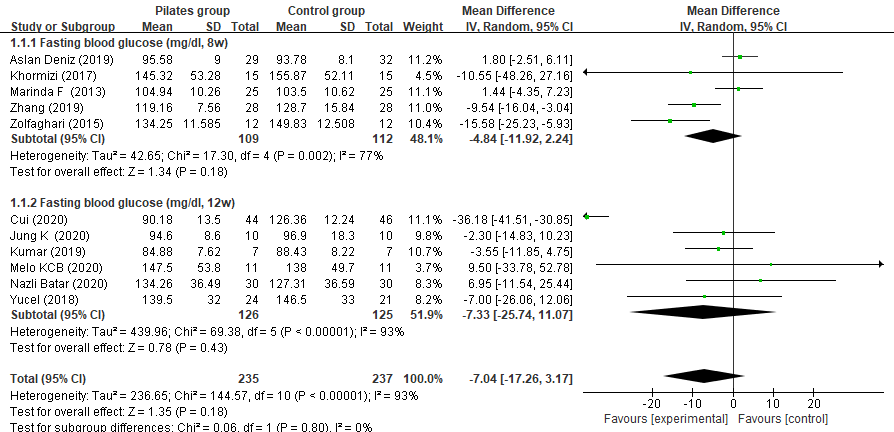


**Supplementary eFigure 3** Meta‐analysis and forest plot and for fasting blood glucose at different intervention doses.


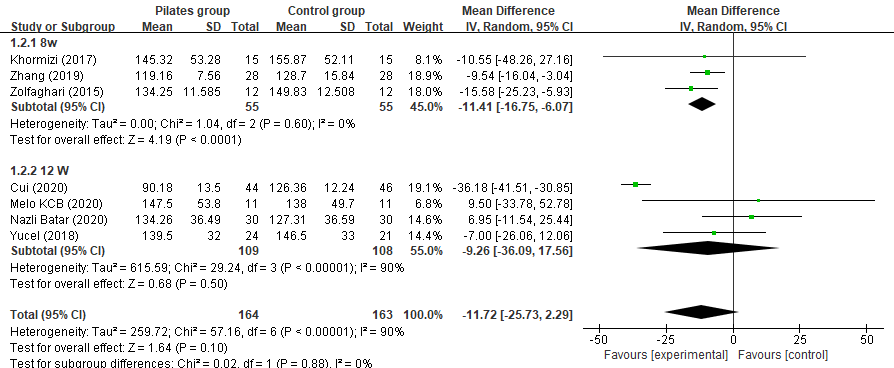


**Supplementary eFigure 4** Meta‐analysis and forest plot and for fasting blood glucose at different intervention doses among diabetes patients.


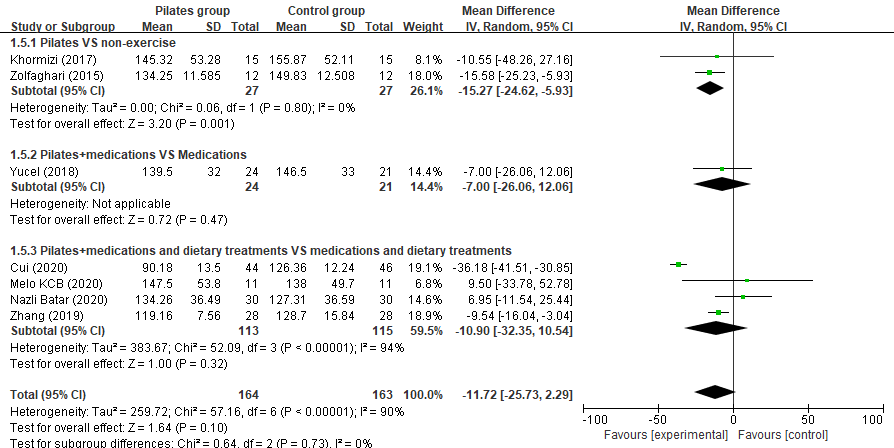


**Supplementary eFigure 5** Meta‐analysis and forest plot and for fasting blood glucose in diabetes patients.

**
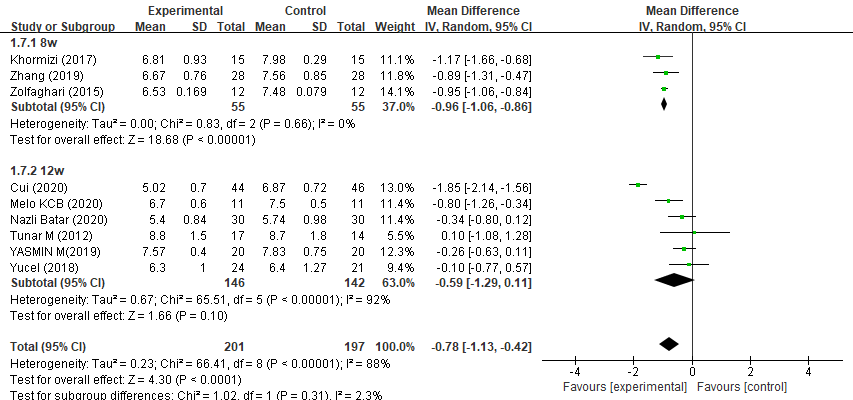
**

**Supplementary eFigure 6** Meta‐analysis and forest plot and for HbA1c at different intervention doses among diabetes patients.
